# Membrane-mediated action of the endocannabinoid anandamide on membrane proteins: implications for understanding the receptor-independent mechanism

**DOI:** 10.1038/srep41362

**Published:** 2017-01-27

**Authors:** Djalma Medeiros, Laíz da Costa Silva-Gonçalves, Annielle Mendes Brito da Silva, Marcia Perez dos Santos Cabrera, Manoel Arcisio-Miranda

**Affiliations:** 1 Laboratório de Neurobiologia Estrutural e Funcional (LaNEF), Departamento de Biofísica, Escola Paulista de Medicina, Universidade Federal de São Paulo, São Paulo, SP, Brasil; 2 Curso de Filosofia, Faculdade de São Bento, São Paulo, SP, Brasil; 3 Departamento de Química e Ciências Ambientais, IBILCE, Universidade Estadual Paulista, São José do Rio Preto, SP, Brasil

## Abstract

Endocannabinoids are amphiphilic molecules that play crucial neurophysiological functions acting as lipid messengers. Antagonists and knockdown of the classical CB1 and CB2 cannabinoid receptors do not completely abolish many endocannabinoid activities, supporting the idea of a mechanism independent of receptors whose mode of action remains unclear. Here we combine gramicidin A (gA) single channel recordings and membrane capacitance measurements to investigate the lipid bilayer-modifying activity of endocannabinoids. Single channel recordings show that the incorporation of endocannabinoids into lipid bilayers reduces the free energy necessary for gramicidin channels to transit from the monomeric to the dimeric conformation. Membrane capacitance demonstrates that the endocannabinoid anandamide has limited effects on the overall structure of the lipid bilayers. Our results associated with the theory of membrane elastic deformation reveal that the action of endocannabinoids on membrane proteins can involve local adjustments of the lipid/protein hydrophobic interface. The current findings shed new light on the receptor-independent mode of action of endocannabinoids on membrane proteins, with important implications towards their neurobiological function.

Endocannabinoids are amphiphilic molecules which are synthesized from membrane phospholipids within the nervous system. In association with their G-protein coupled receptors they form the so-called endocannabinoid system^1^,^2^,^3^,^4^,^5^. This lipid system, alone or in combination with other signaling systems, is involved in a number of fundamental neurophysiological processes, including neurogenesis, reward, cognition, learning, memory acquisition, and pain sensation^6^,^7^,^8^. Disorders of the endocannabinoid system have been correlated with several neuro-inflammatory diseases such as Alzheimer, Parkinson, Huntington, Multiple Sclerosis, and Amyotrophic Lateral Sclerosis^9^,^10^,^11^,^12^. Also, the hyperactivity of this system is associated to metabolic disorders and obesity^13^.

The best studied endocannabinoids are the N-arachidonylethanolamide (AEA or anandamide) and the 2-arachidonoylglycerol (2-AG) (Fig. 1a). They consist of an amide or an ester head, conjugated with an arachidonyl chain (20 C with 4 unsaturations, ω6). Although it is well accepted that their (patho)physiological activities mainly occur by binding to cannabinoid receptors or TRP channels^14^,^15^, AEA and 2-AG can also produce effects that are not mediated by these mechanisms^16^,^17^,^18^,^19^,^20^,^21^,^22^. In fact, it has been shown that the endocannabinoids can modulate the activity of many membrane proteins even in the presence of antagonists of their classical receptors. However, the molecular basis underlying the receptor-independent mechanism remains poorly understood.

The modulation of the activity of different types of membrane proteins with diverse amino acid sequences and membrane topologies suggests the lack of a specific binding site for the endocannabinoids into those proteins. In consonance, a common mechanism arising from changes in lipid bilayer properties that modifies the energy of hydrophobic coupling between protein and their host bilayer could be hypothesized. Indeed, several studies have shown that different classes of amphiphiles can partition into the lipid bilayer and regulate the membrane protein function by altering the physico-chemical properties of the lipid bilayer^23^,^24^,^25^.

The insertion of a membrane protein into the hydrophobic environment of the lipid bilayer is associated with an energy cost, the bilayer deformation energy (Δ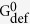), which is related to the adjustments of the lipid bilayer to the membrane protein hydrophobic portion. Andersen and others^26^,^27^,^28^,^29^,^30^,^31^,^32^,^33^,^34^, using the theory of membrane elastic deformation, have extensively analyzed the association between different conformations of a membrane protein and Δ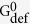 (for review see Lundbaek *et al*.^35^). The free energy cost for the conformational change of a membrane protein from state I to state II (Δ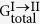) depends on contributions from the membrane protein (Δ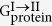) and the bilayer (Δ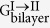). Thus, amphiphilic molecules that change the physico-chemical properties of the lipid bilayer as elasticity, thickness, and intrinsic curvature, may alter the conformational distribution of membrane proteins by changing Δ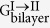.

Here we combined single channel gramicidin A recordings and membrane capacitance measurements to study the bilayer-modifying properties of endocannabinoids. Our observations reveal that, in a model free of cannabinoid receptors, these amphiphilic molecules may reduce the free energy for a membrane protein to transit between different conformational states. Membrane capacitance results show that the insertion of endocannabinoids into model phospholipid bilayers has limited effects on the bilayer’ thickness, supporting the idea of local modifications. Together, these data imply on the proposal of a new mode of action for the endocannabinoids, independent of receptors and based on their effects on membrane/protein hydrophobic interface. This membrane-mediated action may serve as a good model for understanding the endocannabinoid’s receptor-independent effects observed in neurobiological and other systems.

## Results

### AEA modifies gA single channel activity

**Figure 1:**
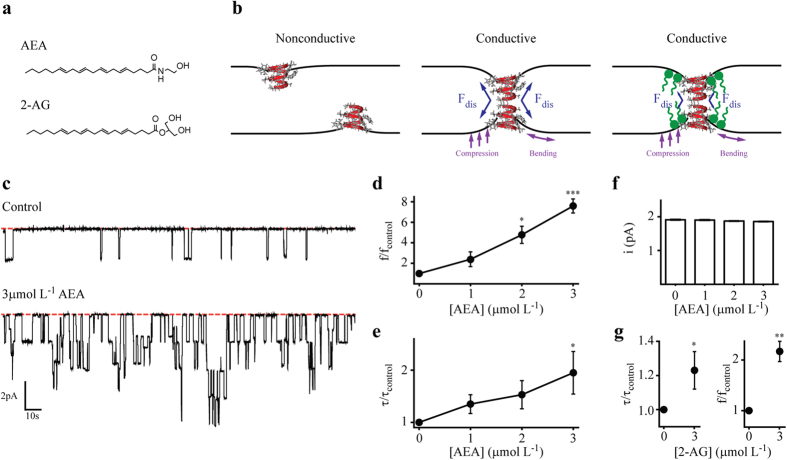
AEA and 2-AG increase gA single channel activity in DOPC bilayers. **(a)** Molecular structure of the endocannabinoids N-arachidonylethanolamide (AEA) and 2-arachidonoylglycerol (2-AG). **(b)** Schematic representation of nonconductive and conductive states of gA channels. A transition from the nonconductive to the conductive state determines a local deformation of the lipid bilayer. In opposition, the lipid bilayer exerts a disjoining force (F_dis_) on the gA channels. The partition of amphiphiles at the lipid/protein hydrophobic interface can alter the lipid bilayer properties and thus the F_dis_. **(c)** gA single channel representative current traces in the absence (top) and in the presence of (bottom) 3 μmol L^−1^ AEA. Red dashed lines indicate the nonconductive state of gA channels. **(d–f)** Concentration-dependent effects of AEA on gA channels: appearance frequency (**f**), open lifetime (τ), and single channel currents (**i**). Data is shown as mean ± s.e.m. (n = 4). P < 0.05, two-way ANOVA, Bonferroni post test. **(g)** Effects of 3 μmol L^−1^ 2-AG on τ and f of gA channels. Data is shown as mean ± s.e.m. (n = 3). P < 0.001, Student’s t-test.

To test the hypothesis that endocannabinoids are bilayer-modifying amphiphiles, we first measured the effects of AEA on the single channel activity of gramicidin A (gA) channels. A gA monomer is a 15-amino acids peptide, right-handed β-helical, single-stranded, with 1.3 nm length^36^. In a bilayer, two nonconductive gA monomers from opposite leaflets can be brought into contact and form the conductive gA channel by establishing formyl end-bonds via six head-to-head hydrogen bonds^35^. The gA channel formation involves a local deformation of the lipid bilayer which occurs to match the hydrophobic length of the channel (Fig. 1b). There is an energy cost associated with this phenomenon. In opposition, the lipid bilayer responds exerting a disjoining force (F_dis_) on the gA channel, where the magnitude of F_dis_ is mainly determined by the elastic properties of the lipid bilayer. The kinetics of gA channel formation and dissociation is described by the reaction


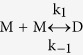


where M and D represent the gA monomer and dimer, respectively, and k_1_ and k_−1_ are the association and dissociation rate constants, respectively.

AEA is a strong modifier of gA activity in DOPC bilayers (Fig. 1c), increasing the appearance rate of gA channels. To quantify this variation, we measured the effects on channel appearance frequency (f) and open channel lifetime (τ). In a concentration-dependent manner, AEA increases f and τ (Fig. 1d and e, respectively). No change was observed in the gA single-channel current transition amplitudes (Fig. 1f). In addition, the presence of 30 mol% cholesterol on DOPC membranes does not change the effects of AEA on the gA channels (Figure S1), although cholesterol influences the AEA translocation across the membrane^37^. To a lesser extent than AEA, 3 μmol L^−1^ 2-AG also increases f and τ of gA channels (Fig. 1g).

**Figure 2:**
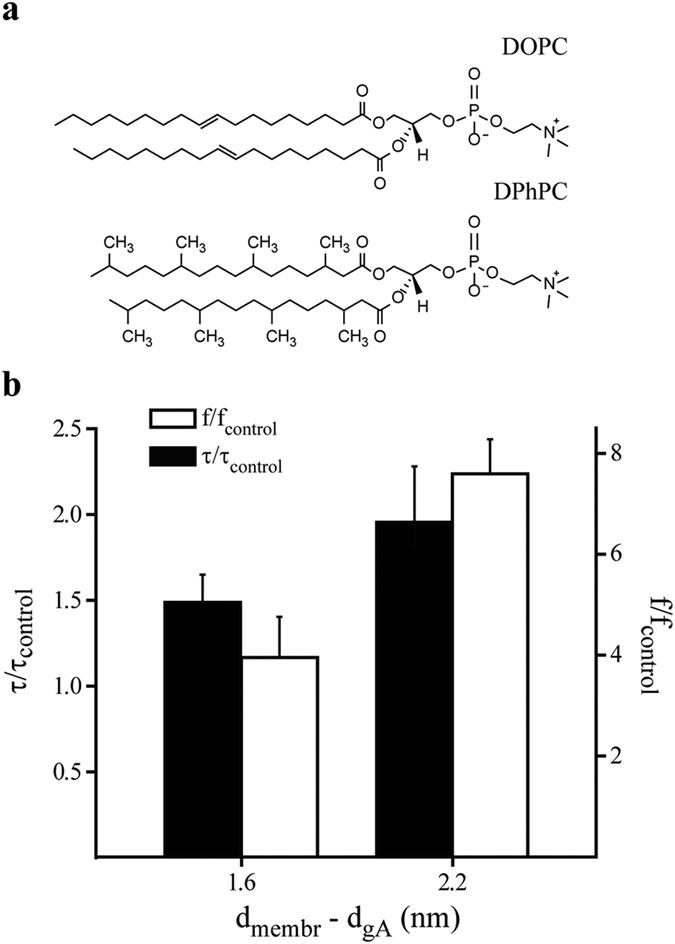
Hydrophobic mismatch-dependent effects of AEA on gA channels. **(a)** Molecular structures of phospholipids used in this study. **(b)** Effects of 3 μmol L^−1^ AEA on gA channel appearance frequency (f) and open channel lifetime (τ) in DOPC and DPhPC bilayers. The x-axis represents the hydrophobic mismatch between gA and the phospholipids used in this study. gA estimated length is based on results from ref. 36. DOPC and DPhPC estimated lengths are based on results from refs.^38^, ^39^, ^40^.

Following, we explored the effects of AEA on the activity of gA channels in lipid bilayers with different hydrophobic lengths to evaluate the effects of different protein/bilayer hydrophobic mismatches. DOPC and DPhPC have, respectively, an estimated hydrophobic length of 4.8 and 4.2 nm^38^,^39^,^40^. AEA also increases f and τ in DPhPC bilayers (Figure S2). However, the magnitude of changes in both parameters is higher for the condition with larger protein/bilayer hydrophobic mismatch, in this case for gA/DOPC bilayer system (Fig. 2). These results may reflect changes on bilayer elasticity^41^.

### Global vs. local membrane modification

**Figure 3:**
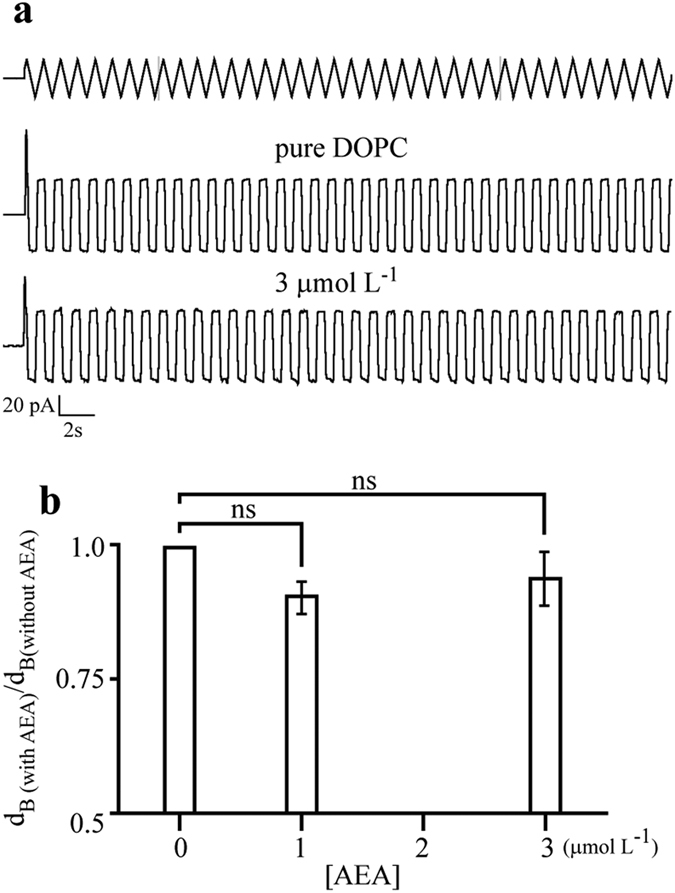
AEA has limited effect on the overall structure of lipid bilayers. **(a)** Representative capacitive current traces without (pure DOPC) and with 3 μmol L^−1^ AEA. Top trace represents the triangular pulse protocol used to obtain the capacitive currents. **(b)** Membrane thickness obtained from membrane capacitance measurements with increasing AEA concentration; ns = not statistically significant.

The above findings raise the following question: If AEA is a bilayer-modifier amphiphile, which are the lipid bilayer properties that it alters ? To answer this question we investigated global changes on lipid bilayers by membrane capacitance measurements, which are mainly determined by the thickness of the membrane. This parameter is largely determined by the lipid constituents of the membrane^42^. Figure 3 shows the effects of AEA on the global structure of DOPC bilayers, indicating that the addition of AEA molecules does not induce significant changes in membrane thickness (Fig. 3b). Thus, these results suggest that global changes on the lipid bilayer cannot account for the observed effects of AEA on the gA channel activity.

Next, we evaluated the effects of AEA on gA channel kinetic parameters, k_1_ and k_−1_. Changes on these parameters have been associated with local deformation of lipid bilayers^33^,^43^. Considering that f and τ can be related to gA channel kinetics by the following relations: f/f_control_ = k_1_/k_1,control_ and τ_control_/τ = k_−1_/k_−1,control_, and the equilibrium constant K_eq_/K_eq,control_ = (f × τ)/(f_control_ × τ_control_), we can obtain the changes in gA channel energetics as


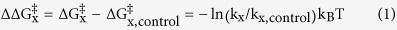



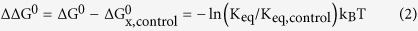


where ΔΔ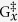 and ΔΔG^0^ are the AEA-induced difference in the activation and equilibrium free energies relative to the control (i.e, in the absence of AEA), respectively. The subscript x denotes 1 or −1, k_B_ is the Boltzmann’s constant, and T is the temperature (Kelvin).

**Figure 4:**
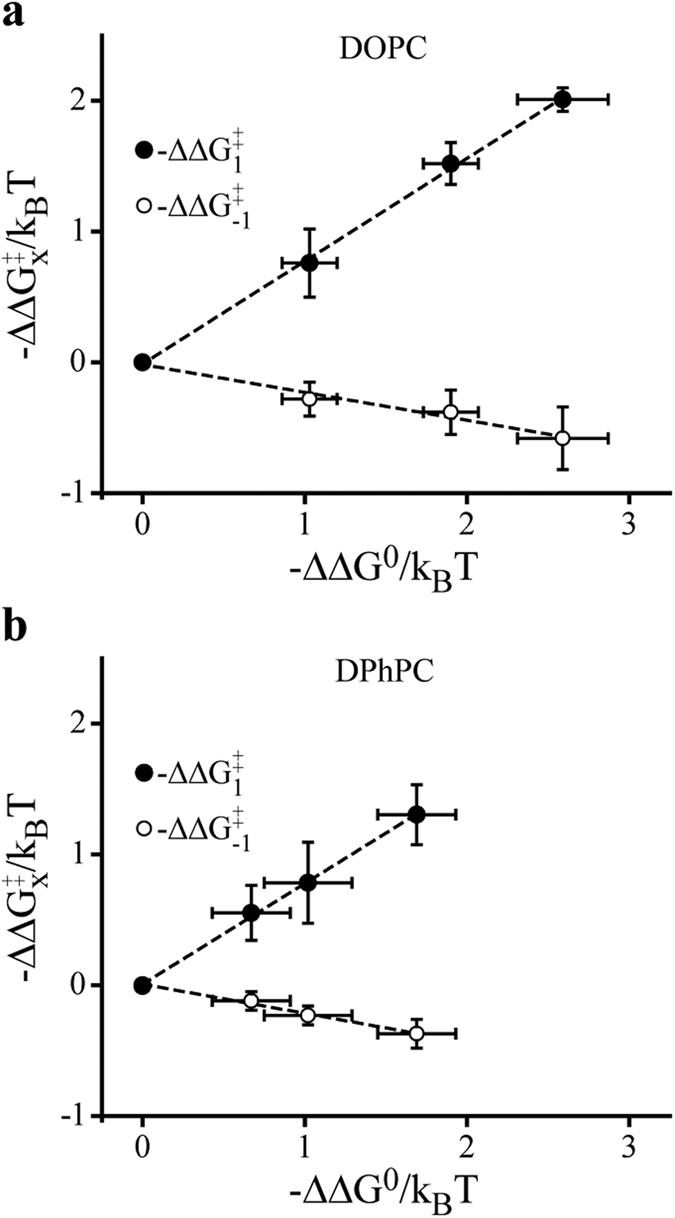
AEA shows linear rate-equilibrium energetic effects on gA channels. Concentration-dependent effects of AEA on gA channel energetics expressed as −ΔΔ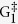 or −ΔΔ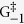 vs. −ΔΔG^0^ in DOPC **(a)** and DPhPC **(b)** bilayers. Data is shown as mean ± s.e.m. (n > 3).

We observed that AEA-induced changes on gA channel activity is a linear function of the relation between −ΔΔ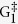 vs. −ΔΔG^0^, with high r-values (DOPC = 0.997 and DPhPC = 0.997) (Fig. 4). These results can be interpreted as a reduction in the activation energy for the monomeric subunit association (Δ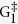) and an increase in the activation energy for the dimer dissociation (Δ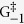). These observations are consistent with the fact that modifications in the energy of a reaction lead into a linear rate-equilibrium relation between the activation free energy (ΔG^‡^) and the equilibrium free energy (ΔG^0^)^33^,^43^.

Using the theory of membrane elastic deformation (see Lundbaek *et al*., for a review)^35^, several works have shown that the energetic cost associated with amphiphile-induced alterations in gA channels (ΔG^0^) is primarily due to changes in bilayer deformation energy (Δ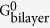), which is related with local deformations (bending or compression) into the lipid/protein hydrophobic interface. In short, the changes in Δ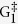 are due to changes in Δ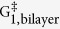, and the slope α of the relation between −ΔΔ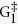 vs. −ΔΔG^0^ can be written as^43^


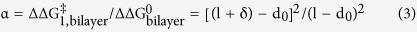


where δ is the distance of ~0.16 nm that separates the gA monomers in the nonconductive state^33^, l is the gA hydrophobic length, and d_0_ is the bilayer hydrophobic length.

We found α equal to 0.776 and 0.775 for DOPC and DPhPC bilayers, respectively, in good accordance with the previous reported value of 0.83 for many structural diverse amphiphiles^43^.

## Discussion

Our understanding of the mode of action of amphiphilic molecules on membrane proteins remains limited. Two mechanisms that do not exclude each other have been proposed. A direct mechanism that requires the binding of the molecules at specific sites within the protein and a nonspecific mechanism whose mode of action involves the perturbation of the host lipid bilayer. For endocannabinoids, the most widespread mode of action involves their binding to G-coupled receptors or TRP channels^14^,^15^. However, this mechanism does not fully explain the endocannabinoid action on membrane proteins. For example, AEA reduces the ionic currents of many voltage-gated ion channels even in the presence of antagonists of their classical CB_1_ and CB_2_ receptors^16^,^17^,^18^,^19^,^21^,^22^. The action of endocannabinoids that alters the function of multiple membrane proteins can indicate the existence of a common, receptor-independent, and more general mechanism.

Thus, in this study we used an inter-disciplinary approach to address the nature of the receptor-independent mode of action of endocannabinoids. Electrophysiology techniques provided the basis to characterize the lipid bilayer-modifying activity of these amphiphiles and allowed to integrate the present results with the well-established theory of membrane elastic deformation. Our experimental strategy of using gA single channel and membrane capacitance measurements to have an energetic examination of the process has followed the studies by Andersen and Lundbaek^33^,^44^,^45^. This strategy has been successfully employed to analyze the promiscuous action of diverse structural amphiphilic drugs and has shown good correlation with the action of these drugs with more complex membrane proteins.

Our results of gA single channel show that AEA increases the channel appearance frequency (f) and the average lifetime (τ) of the channel open state. Additionally, the observed effects are more potent in membranes with higher hydrophobic mismatch. Indeed, 3 μmol L^−1^ AEA increased f by a factor of 7.2 in DOPC (18:1) versus 4 in DPhPC (16:0) bilayers. τ was increased by a factor of 1.8 in DOPC versus 1.4 in DPhPC.

Following the footsteps of the theory of membrane elastic deformation, the changes in f and τ indicate that AEA increases the bilayer elasticity (bending or compression), reducing the bilayer deformation energy associated with channel formation^35^. The correlation between channel kinetics and energetics shows that the action of AEA on gA channels has a linear rate-equilibrium relation with a slope determined by ΔΔ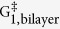/ΔΔG^0^. This result provides strong evidence for the hypothesis that the effects of AEA are mediated by a more general and nonspecific mechanism associated with locally adjusting the bilayer hydrophobic thickness to match the channel length. Similar results with other amphiphilies, including curcumin^32^, DHA^46^, and PIP_2_^47^, have also been interpreted to mean that their effects are not due to a direct modification of the membrane protein properties, but are related to a nonspecific modification of the bilayer physical properties at the bilayer/protein hydrophobic interface. In fact, the actions of molecules that alter gA channel function by other mechanisms than changes in bilayer elasticity are expected to deviate from the linear rate-equilibrium relation. Thus, if a direct interaction of AEA with the protein had occurred, this would give rise to additional ΔΔ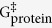 contributions to ΔΔ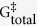 (considering Δ
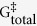
=Δ
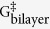
+Δ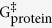), and their effects would deviate from the linearity. The fact that AEA does not produce detectable changes on the amplitude of gA ionic currents largely agrees with the theoretical arguments presented above and also goes against a direct binding within the protein^46^.

We consequently explored the effects of AEA on membrane structure for a possible physical nature of the mechanism. Although there are many evidences that endocannabinoids can induce changes on lipid bilayer fluidity^37^,^48^, this phenomenon cannot account for changes in the energetics of membrane protein conformational transitions^49^. Our capacitance measurements show that AEA molecules produce limited effects on bilayer overall structure. However, an established concept that we cannot rule out is that changes on lateral pressure profile of lipid bilayers could lead to protein conformational transitions^50^,^51^. Recently, Ingólfsson *et al*.^52^ have characterized the interaction of some phytochemicals with lipid bilayers and provided evidences that changes on the lateral pressure profile of the lipid bilayer by these amphiphilic molecules appears to be involved on conformational transitions of gramicidin and other channels. Future experiments should explore this possibility for the endocannabinoids in order to obtain a greater molecular view of this mechanism.

Finally, it is important to note that our study does not preclude membrane protein modulation by endocannabinoid’s direct binding to G-coupled receptors or TRP channels. However, our data also argue in favor of an alternative mode of action of endocannabinoids on membrane proteins, independent of receptors and based on their ability to alter the bilayer elastic properties.

## Concluding Remarks

Endocannabinoids are known to exert many neurophysiological functions and are also involved in neuropathological conditions. By applying electrophysiology studies we provide direct evidence of a membrane-mediated action for the endocannabinoids on membrane proteins. The lack of evidence for a direct binding on gA and the limited changes on the overall structure of the lipid bilayer supports the hypothesis of a local action for the endocannabinoids. This is also sustained by an energetic analysis following the theory of membrane elastic deformation that shows a linear rate-equilibrium relation for the action of AEA on gA single channel activity. Given the remarkable agreement between our endocannabinoids’ data and results from other amphiphiles, this study proposes a mechanistic model for the receptor-independent action of the endocannabinoids. By changing the lipid bilayer elastic proprieties, the endocannabinoids can modulate the activities of embedded proteins. Thus, our findings may benefit other endocannabinoid’s signaling studies by providing for the first time a molecular explanation of the receptor-independent mode of action suggested by several studies. However, the deciphering of the membrane-mediated action of endocannabinoids in more complex neurobiological systems with all possible endocannabinoid’s targets will be an experimental challenge in the next years, as well as the understanding of the endocannabinoid’s structural features and the functional interaction between the receptor-dependent and receptor-independent modes of action of these amphiphilic molecules. At the same time, understanding this complex scenario will define specific functions for each mode of action and may provide useful templates for the design of new therapeutics that mimic endocannabinoids.

## Experimental Section

### Materials

We purchased the following lipids from Avanti Polar Lipids, Inc. (Alabama, USA): 1,2-diphytanoyl-sn-glycero-3-phosphocholine (DPhPC) and 1,2-dioleoyl-sn-glycero-3-phosphocholine (DOPC). Gramicidin A (gA), Arachidonylethanolamide (AEA), 2-Arachidonyl glycerol (2-AG), cholesterol, and all other chemicals were purchased from Sigma-Aldrich (Missouri, USA). gA was further purified by high performance liquid chromatography (HPLC)^53^ using the Vydac C18 column and a gradient of 10 to 100% acetonitrile.

### Electrophysiology experiments

Planar lipid bilayers were formed following the “painting method”^54^ in a custom-made bilayer setup fabricated in acrylic with two compartments – cis (front) and trans (posterior) – of 4 mL capacity. These two compartments were separated by a thin Polyethylene film that contained one aperture with a diameter of ~150 μm. For the bilayer formation, the desired lipid solution (25 mg mL^−1^ stock solution) was spread across the polyethylene film aperture. The compartments cis and trans were filled with an electrolyte solution composed of 10 mmol L^−1^ HEPES and 1 mol L^−1^ KCl, pH 7.4. All experiments were carried out at room temperature (23 ± 2 °C). The electrical access to the bath solutions was made by a pair of Ag/AgCl electrodes. The cis compartment was held at ground and the trans compartment was clamped at chosen potential by a patch-clamp amplifier (PC-One, Dagan Corporation, Minnesota, USA) configured in voltage-clamp mode. The amplifier was connected to a data acquisition board (DigiData 1440 A, Molecular Devices, USA) set at a sampling frequency of 1 kHz. Data acquisition was carried out using Axoscope 10.2 (Molecular Devices, USA).

For single-channel recordings, after formation of a stable lipid bilayer, small volumes (0.5 μL) of a 300 nmol L^−1^ gA solution in ethanol were added to both compartments of the bilayer setup until one gA channel could be observed in the bilayer. After each addition of gA, the bath solutions were stirred for at least two minutes. To measure the single-channel conductance of gA channels, current-versus-time traces were recorded while a voltage of −100 mV was applied across the lipid bilayers. The frequency of gA channel appearance (f) was determined from the number of channel events divided by the total recording time. Open state single-channel gA lifetimes (τ) were obtained by fitting survivor histograms with single-exponential function as


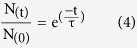


where N_(t)_ is the number of channels with lifetime longer than the time t.

For membrane capacitance measurements, an arbitrary waveform generator (33521 A Agilent Technologies, USA) was connected to the voltage input channel of the amplifier and the membrane capacitance (C_m_) was obtained using an auxiliary triangular voltage pulse of 1 Hz frequency and 100 mV_p-p_ amplitude. C_m_ values were determined as^55^


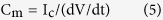


where I_c_ is the membrane capacitive current and dV/dt is the sweep rate of the auxiliary triangular voltage pulse.

The bilayer thickness (dB) was then obtained as


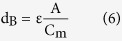


where ε is the material dielectric constant, and A is the membrane area.

Aliquots of endocannabinoid ligands (5 mmol L^−1^ stock solutions in ethanol) were added, at the desired concentration, to both compartments of the lipid bilayer setup. Final ethanol concentration was less than 0.1% and showed no change, per se, on the electrical parameters of the lipid bilayers or gA single channel activity.

gA single channel activity and the membrane capacitance were analyzed with the Clampfit software 10.4 (Molecular Devices, CA, USA). Statistical analyses were performed with GraphPad Prism 6.0 (GraphPad Software, Inc., CA, USA) and the significance (p < 0.05) was determined by two-way ANOVA test and Bonferroni’s method. The results are shown as mean ± s.e.m.

## Additional Information

**How to cite this article**: Medeiros, D. *et al*. Membrane-mediated action of the endocannabinoid anandamide on membrane proteins: implications for understanding the receptor-independent mechanism. *Sci. Rep.* **7**, 41362; doi: 10.1038/srep41362 (2017).

**Publisher's note:** Springer Nature remains neutral with regard to jurisdictional claims in published maps and institutional affiliations.

## Supplementary Material

Supplementary Information
