# Supplementary material for: Membrane-mediated action of the endocannabinoid anandamide on membrane proteins: implications for understanding the receptor-independent mechanism
Source: Sci Rep. 2017 Jan 27;7:41362. doi: 10.1038/srep41362 (PMC5269673; doi:10.1038/srep41362)
Supplement: Supplementary Information [file srep41362-s1.pdf]

Membrane-mediated action of the endocannabinoid anandamide on membrane proteins: implications for understanding the receptor-independent mechanism

Djalma Medeiros,[†¶#] Laíz da Costa Silva-Gonçalves,[†#] Annielle Mendes Brito da Silva,[†] Marcia Perez dos Santos Cabrera,[§] Manoel Arcisio-Miranda[†*]

[†] Departamento de Biofísica, Escola Paulista de Medicina, Universidade Federal de São Paulo, São Paulo, SP, Brasil;

[¶] Curso de Filosofia, Faculdade de São Bento, São Paulo, SP, Brasil;

[§] Departamento de Química e Ciências Ambientais, IBILCE, Universidade Estadual Paulista, São José do Rio Preto, SP, Brasil

[#] These authors contributed equally to this work.

Supplementary Information:

Supplementary figures: Figures S1 – S2

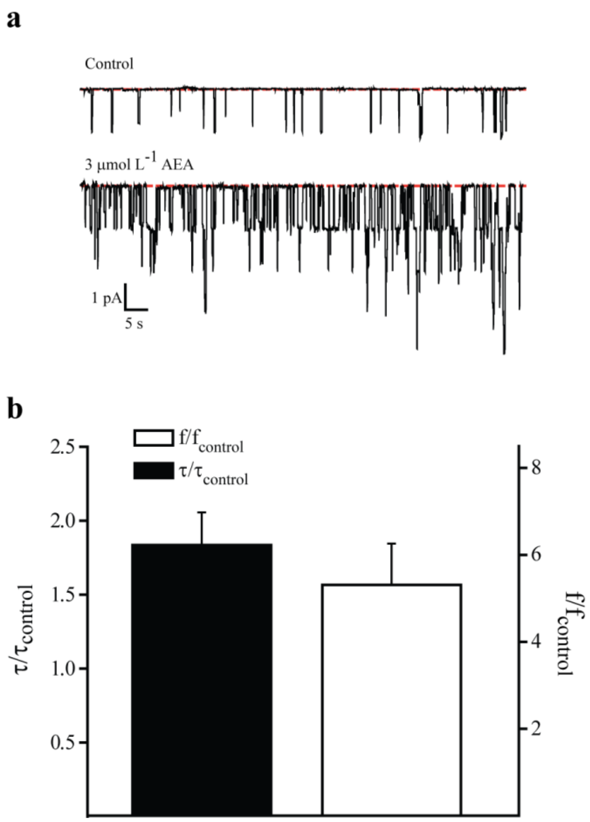

Figure S1: AEA increases gA single channel activity in DOPC/Cholesterol bilayers. (a) gA single channel representative current traces without (top) and with (bottom) 3 μmol L^{-1} AEA. Red dashed lines indicate the nonconductive state of gA channels. **(b)** Relative effects of 3 μmol L^{-1} AEA on τ and f of gA channels. Data is shown as mean ± s.e.m. (n = 3).

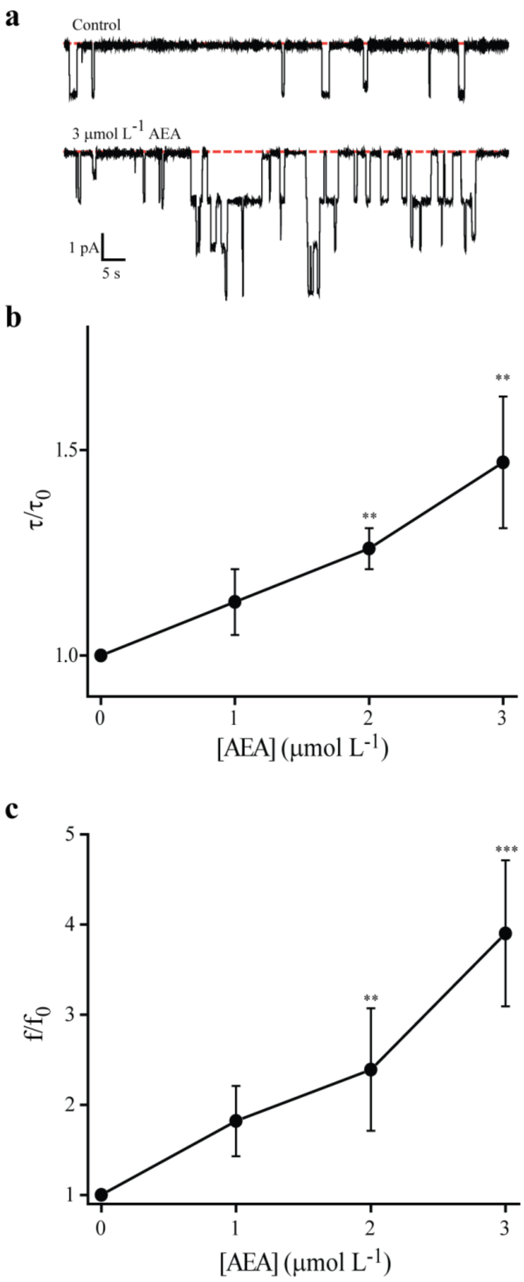

Figure S2: AEA increases gA single channel activity in DPhPC bilayers. (a) gA single channel representative current traces without (top) and with (bottom) 3 μmol L^{-1} AEA. Red dashed lines indicate the nonconductive state of gA channels. **(b)** Concentration-dependent effects of AEA on the open lifetime (τ). **(c)** Concentration-dependent effects of AEA on the appearance frequency (f). Data is shown as mean ± s.e.m. (n = 3). P value was determined using Bonferroni´s method ($P < 0.05$).
